# Long-Term Tennis Participation and Self-Efficacy in Older South Korean Male Adults: A Qualitative Study

**DOI:** 10.3390/healthcare14101308

**Published:** 2026-05-12

**Authors:** Youngjik Lee

**Affiliations:** Department of Sports Industry, Korea National University of Transportation, Daehak-ro 50, Chungju 27469, Republic of Korea; youngjik.lee@ut.ac.kr

**Keywords:** self-efficacy, tennis participation, older adults, healthy aging, qualitative research

## Abstract

**Background**: Self-efficacy is an important psychological factor for healthy aging, but how long-term sport participation builds self-efficacy in older adults is not fully understood. This study explored how playing tennis for many years shapes self-efficacy in older South Korean adults and identified the key mechanisms. **Methods**: In-depth interviews were conducted with 11 older male adults (aged 65–75 years) who had played tennis for 15–25 years and remained active at least twice per week. Participants were recruited from tennis clubs in South Korea through purposive sampling. Data were analyzed using Braun and Clarke’s reflexive thematic analysis. **Results**: Four themes emerged. (1) Mastery Through Progressive Achievement: gradual skill development and competitive success were perceived to support confidence that extended beyond the tennis court; (2) Social Embeddedness and Collective Efficacy: peer encouragement and observing similar others succeed were described as supporting participants’ belief in their own capabilities; (3) Physical Vitality as Confidence Foundation: sustained physical fitness and functional independence derived from tennis participation were perceived to support broader self-confidence in daily life; and (4) Mental Resilience and Cognitive Engagement: the strategic demands of tennis and its stress-relieving effects may contribute to psychological resilience and a continued sense of purpose in later life. **Conclusions**: Long-term tennis participation was perceived to support self-efficacy through multiple interconnected pathways consistent with Bandura’s social cognitive theory. These findings suggest that structured, community-based tennis programs may contribute to supporting psychological well-being and promoting healthy aging in rapidly aging societies.

## 1. Introduction

As economic growth and improvements in medical technology have elevated the standard of living worldwide, the global older adult population is growing at a rapid pace [1,2]. As of 2020, about 9.3% of the global population was aged 65 or older. By 2050, this will increase to over 16% globally [3]. South Korea is also one of the world’s fastest aging countries.

Population aging, while bringing many positive outcomes for public health and economic development, also poses certain psychological challenges [4]. Currently, most mental health systems are developed to respond to the needs and problems of the general population. However, very few resources and services are designed for older adults [5]. Furthermore, many of today’s social environments do not offer opportunities for older adults to discover meaning, purpose and social connection and can therefore contribute to and worsen psychological distress [6].

Many older adults experience periods of social isolation caused by retirement, physical disability or bereavement. A recent meta-analysis by social psychologist Holt-Lunstad found that social isolation has been linked with a 29% increased risk of mortality comparable to other major health risks, including obesity and smoking [7]. The association between social isolation and health may be indirect through fewer healthy behaviors, greater physical and psychological stress and less access to social and practical resources [8].

There is some evidence that some technologies intended to reduce social isolation can have a positive effect, mostly by enhancing existing social relationships rather than replacing them [8]. However, there are considerable limitations in the potential of digital communication technologies to support contact between older people and family and friends, particularly for the oldest age groups, because of a lack of access to technology and skills to use it.

In psychological terms, self-efficacy refers to an individual’s perceived capability to perform the actions necessary to attain desired outcomes within a specific domain or context [9]. Belief in self-efficacy is one of the psychological factors of healthy aging [10]. Several studies have shown the relevant influence of self-efficacy in older age on several different aspects of well-being [10]. Positive physical health outcomes such as high physical functional status, less disability and health-enhancing behaviors of older people are associated with high self-efficacy [10,11]. In a recent paper, Rimmele et al. [12] discussed this cognitive aspect of self-efficacy. Self-efficacy has also been found to decrease before a decline in health is perceived, and thus may be causally related to health [10].

Among older adults, self-efficacy has been connected to better thinking skills, emotional strength, and the ability to handle the challenges that come with aging [12,13,14]. Importantly, self-efficacy and healthy behaviors support each other: when people believe they can succeed, they are more likely to engage in healthy behaviors, and those successes then make their beliefs even stronger [15,16]. For this reason, understanding how staying active in specific activities builds and keeps these beliefs is an important part of research on healthy aging.

Nevertheless, relatively little research has addressed how long-term involvement in a single sport, distinct from general physical activity, shapes self-efficacy. The majority of existing studies have relied on short-term interventions or cross-sectional designs, and as a result, the longitudinal processes through which continued sport participation influences efficacy beliefs remain insufficiently explored [17]. In addition to promoting physical and mental health, involvement in physical activity can be a very effective way to promote self-efficacy in older adults. Physical activity provides opportunities for older adults to experience mastery, identified by Bandura [9] as one of the major sources of self-efficacy. Involvement in organized physical activity has also been shown to enhance self-efficacy in older adults in a number of domains [17].

While many sports have advantages for ‘senior’ sports participation, tennis offers unique qualities that make it particularly well-suited for older adults. It is a lifetime sport that can be played at various levels with modifications for fitness and skill. In addition to social connection and competitive drive, tennis requires quick decision making, strategic thinking and movement. While there is some research on tennis with older adults, results indicate positive changes in physical function, cardiovascular fitness and overall quality of life [18,19].

Most research examining tennis and aging has taken a short-term intervention or cross-sectional approach examining a variety of physical and psychological characteristics of older adult tennis players at one point in time [20,21]. However, no research has investigated the effect of continuous participation in tennis on enhancing self-efficacy across adulthood or the mediating variables that might explain how participation in tennis can have a positive effect on self-efficacy as adults age.

South Korea is undergoing one of the most rapid demographic transitions of any developed country; the proportion of older residents is rising at a faster pace than any other developed nation [22]. Keeping quality of life and psychological well-being high during such rapid changes is a considerable challenge for South Korean older adults. The number of older adults participating in tennis is growing rapidly in South Korea, with tennis increasingly recognized as an accessible and socially engaging activity for this population [23].

This study aims to address these gaps by exploring the relationship between long-term tennis participation and self-efficacy in South Korean older adults through a qualitative approach. Qualitative research methods are particularly valuable for investigating these complex psychological processes, as they allow for in-depth exploration of lived experiences and the meanings individuals attribute to their activities [24].

Specifically, this research seeks to: (1) examine how older adults who have participated in tennis for extended periods perceive the influence of tennis on their self-efficacy; (2) identify the specific mechanisms through which tennis participation may enhance or maintain self-efficacy in later life; and (3) explore how tennis participation relates to broader aspects of healthy aging and well-being in the South Korean cultural context.

By addressing these research questions, this study contributes to the growing body of literature on physical activity and psychological well-being in older adults while providing practical insights for developing interventions to promote healthy aging. Understanding how long-term engagement in tennis influences self-efficacy can inform the design of physical activity programs that maximize psychological benefits for aging populations.

## 2. Materials and Methods

### 2.1. Research Design

This study utilized qualitative research through semi-structured interviews to investigate the experiences of older adults involved in long-term tennis participation. More specifically, the study used a theoretically informed qualitative approach, applying Braun and Clarke’s reflexive thematic analysis as the main analytical framework. Qualitative methodology was selected as most appropriate for investigating the complex, subjective nature of self-efficacy beliefs and their relationship with sustained physical activity engagement [25]. This method was chosen because reflexive thematic analysis is well-suited to finding common patterns across participants’ responses, while also allowing the researcher to play an active and reflective role in interpreting the data. This approach allowed for in-depth exploration of participants’ perspectives, meanings, and experiences. The study was grounded in an interpretivist paradigm, acknowledging that reality is socially constructed and that understanding participants’ subjective experiences is central to knowledge generation [26]. This philosophical stance aligns with the study’s aim to understand how older adults themselves perceive and interpret the relationship between their tennis participation and self-efficacy beliefs.

### 2.2. Participants and Sampling

Participants were selected through purposive sampling, a method suitable for qualitative research aiming to gather in-depth cases applicable to the research inquiries [27]. Inclusion criteria were: (1) age 65 years or older; (2) continuous tennis participation for at least 15 years; and (3) current active participation in tennis at least twice weekly. The 15-year threshold was chosen because previous research indicates that at least a decade of consistent sport participation is needed for lasting psychological benefits to develop [18]. A minimum of 15 years has also been widely used in the sport and aging literature to identify those who participate habitually, rather than occasionally. Recruitment occurred through tennis clubs in Chungbuk and Gyeonggi Province, South Korea, between October 2025 and February 2026. Initial contact was made through club administrators, who were given the inclusion criteria by the research team and asked to identify members who appeared to meet these criteria based on their knowledge of club records. A total of 18 individuals were identified as potentially eligible and approached by the researcher. Of these, 15 agreed to participate. However, four were later excluded after screening: two did not meet the minimum years of participation requirement, one did not meet the twice-weekly frequency requirement, and one chose to withdraw after receiving full information about the study. As a result, 11 participants completed the interview process. All participants provided written informed consent before participation.

The final sample consisted of 11 male participants aged 65 to 75 years (mean age = 70 years, SD = 3.3). Years of tennis participation ranged from 15 to 25 years (mean = 19.1 years, SD = 3.7). A total of 13 interviews were conducted. Within the reflexive thematic analysis framework, the adequacy of the data was assessed on an ongoing basis throughout the analytical process, rather than being determined in advance [28]. Thematic saturation, defined as the point at which no new themes or meanings were emerging from the data, was reached after the 11th interview. Two additional interviews were then conducted to confirm this, and no new themes emerged. The two confirmatory interviews are not included in the reported sample, as they did not contribute any additional thematic content [28] (Table 1).

### 2.3. Data Collection

Data were collected through individual, semi-structured interviews conducted in Korean by the researcher. Interview questions were developed based on Bandura’s self-efficacy theory [9] and existing literature on physical activity and aging [29,30]. The interview guide featured open-ended questions aimed at gathering comprehensive narratives regarding participants’ tennis experiences and their self-assessments of their abilities.

The interview guide covered the following main topics: (1) tennis participation history and current practices; (2) perceived physical, mental, and social benefits of tennis; (3) beliefs about personal capabilities and confidence in various life domains; (4) experiences of challenge, achievement, and setbacks in tennis; (5) social relationships and support within tennis communities; and (6) broader impacts of tennis on daily life and aging experiences.

Interviews were conducted at locations convenient for participants, including tennis club facilities and parks, while ensuring privacy and comfort. Each interview lasted approximately 45 to 60 min. All interviews were audio-recorded with participants’ permission and transcribed verbatim.

### 2.4. Data Analysis

Data analysis followed Braun and Clarke’s reflexive thematic analysis approach [31,32], a method that is particularly effective for the identification, examination, and documentation of patterns (themes) within qualitative data. This approach highlights the active role of the researcher in knowledge production and the importance of reflexivity throughout the analytical process.

The analysis comprised six phases: (1) familiarization with data through repeated transcript readings and initial notes; (2) systematic coding of notable features within the dataset; (3) theme identification by systematizing codes into prospective themes; (4) reviewing themes to ensure alignment with coded extracts and the comprehensive dataset; (5) defining and naming themes to encapsulate their essence; and (6) producing a scholarly report with illustrative examples [31,32]. All coding and thematic analysis were carried out by the author. Coding was primarily inductive, meaning themes were allowed to emerge from the data rather than being determined in advance. However, the analysis was also informed by Bandura’s self-efficacy theory [9], which directed the researcher’s attention to concepts such as mastery, vicarious experience, social persuasion, and physiological and emotional states.

Initial codes were generated through close reading of the transcripts and then grouped into candidate themes based on shared meaning. These themes were subsequently reviewed against the full dataset to ensure they were coherent, distinct, and well-grounded in the data. Final theme names were developed to reflect the researcher’s interpretive work rather than simply describing the data. Given that the analysis was conducted by a single researcher, reflexivity was maintained throughout the process through ongoing critical reflection on emerging interpretations [31,32]. Analysis was conducted in Korean to preserve linguistic and cultural nuances. Selected quotations were translated into English by a bilingual researcher and were subsequently back-translated by an independent translator for accuracy and equivalence [33]. Quotations were lightly edited for readability in English while preserving the original meaning. The final English quotations were reviewed by the translation team to ensure that meaning and cultural nuance had been adequately preserved. Any inconsistencies were resolved through deliberation among the translation team members.

### 2.5. Trustworthiness

Trustworthiness in qualitative research within a reflexive thematic analysis framework is not achieved through replication or consensus coding, but through transparency, reflexivity, and the rigor of the interpretive process [26].

Credibility was supported through: (1) prolonged and repeated engagement with the data to develop interpretive depth; (2) member checking, used not to verify a single correct reading but to prompt reflection on whether participants recognized their experiences in the researcher’s interpretations; and (3) situating findings within existing literature to explore resonance rather than to triangulate toward a fixed truth.

Transferability (external validity/generalizability) was enhanced through a comprehensive description, offering extensive details regarding the research context, participants, and findings to enable readers to evaluate the relevance of the results to different environments.

Dependability was addressed through: (1) maintaining a clear audit trail documenting all analytical decisions, including how codes were developed, revised, and organized into themes; and (2) transparent documentation of the analytical process to enable readers to evaluate the interpretive logic underpinning the findings.

Confirmability within a reflexive framework does not imply researcher neutrality or objectivity, but rather transparency about the researcher’s positionality and its influence on interpretation. This was addressed through: (1) sustained critical reflection throughout the analytical process to surface and examine the researcher’s assumptions, prior knowledge, and emerging interpretations; and (2) providing rich participant quotations to allow readers to assess the relationship between the data and the interpretations offered.

### 2.6. Ethical Considerations

The study received ethical approval from the Korean National University of Transportation Institutional Review Board (IRB No.: KNUT-2025-HR-09-25) and was conducted in accordance with the Declaration of Helsinki. All participants provided written informed consent after receiving detailed information about the study purpose, procedures, risks, and benefits. Participants were informed of their right to withdraw at any time without consequence. Confidentiality was maintained through the de-identification of all data, with only the research team having access to identifying information. Audio recordings and transcripts were stored securely in password-protected files.

## 3. Results

Reflexive thematic analysis identified four key themes relating long-term tennis participation to self-efficacy in older Korean adults: (1) Mastery Through Progressive Achievement; (2) Social Embeddedness and Collective Efficacy; (3) Physical Vitality as Confidence Foundation; and (4) Mental Resilience and Cognitive Engagement. Each theme is presented below with supporting quotations from participants.

### 3.1. Theme 1: Mastery Through Progressive Achievement

This theme captures participants’ experiences of building self-efficacy through accumulated achievements and skill development in tennis. Participants consistently described how successfully mastering tennis skills, improving performance, and achieving goals reinforced their beliefs in their capabilities both on and off the court.

Participants emphasized the importance of incremental progress and the satisfaction derived from observable improvement. As one participant (P3) explained:

*When I first started tennis at 50, I could barely hit the ball over the net. But year by year, I got better. Now I can play competitively in my club. Every small improvement—a better serve, a successful backhand—it builds my confidence. And that confidence doesn’t stay on the court. It makes me feel like I can tackle other challenges in life too.*

The structured nature of tennis, with clear markers of progress such as improving technique, winning games or advancing to higher skill groups, provided strong evidence of capability. This tangible feedback appeared particularly important for self-efficacy development. Participant P9 described:

*In tennis, you can see your progress clearly. You win more games, you make fewer errors, your movements become more fluid. This visible improvement is powerful. It proves to yourself that you’re still capable of learning and growing, even in your 70s.*

Several participants compared tennis with other activities, noting that the competitive and skill-based nature of the sport provided unique opportunities for mastery experiences. Participant P1 reflected:

*I also do walking and some stretching exercises, and those are good for health. But tennis is different. In tennis, I am constantly challenged, constantly trying to improve. When I succeed—when I win a match or finally master a difficult shot—the sense of achievement is tremendous. It makes me believe in myself in a way that just walking can’t.*

Participants also described how overcoming tennis-specific challenges were perceived to build resilience and confidence that they experienced as transferring to other life challenges. Participant P7, the oldest participant with 25 years of tennis experience, explained:

*Tennis teaches you to handle setbacks. You miss shots, you lose games, but you keep trying. This persistence builds character. When I face difficulties outside tennis—health issues, family problems—I draw on that same resilience. Tennis has taught me that I can overcome obstacles if I keep working at it.*

The achievement experiences in tennis appeared to create a positive feedback loop, where success bred confidence, which in turn facilitated further engagement and achievement. Participant P6 described this cycle:

*The better I got at tennis, the more confident I felt. And the more confident I felt, the more willing I was to try new things—not just in tennis, but in other areas of my life. I started taking computer classes, I volunteered for community activities. Tennis opened up this whole new sense of possibility.*

This sense of achievement through goal attainment was echoed by a participant who had competed at the club level for decades. Participant P11, who first encountered tennis as a spectator during the 1986 Asian Games but began regular participation years later, reflected on the confidence derived from competitive success:

*In the past, within the club I often played the role of leader. When we competed according to the proper format, I almost always placed. And I gain confidence in everything—in other aspects of daily life as well.*

Similarly, another long-term participant highlighted the uniquely challenging yet rewarding nature of tennis as a driver of ongoing mastery motivation. Participant P8, with nearly 25 years of tennis experience, explained:

*Tennis is very difficult compared to other sports. But at the same time, there seems to be no sport as fun and thrilling as this one. For example, when I hit a passing shot and the opponent cannot reach it—the satisfaction from landing the ball exactly where you intended is tremendous.*

### 3.2. Theme 2: Social Embeddedness and Collective Efficacy

This theme reflects the profound influence of social relationships and community belonging within tennis networks on participants’ self-efficacy. Participants described how the social dimensions of tennis participation provided emotional support, encouragement, and vicarious experiences that strengthened their beliefs in their capabilities.

The tennis community emerged as a primary source of social connection and belonging for participants. Participant P2 explained:

*My tennis friends are like a family. We meet several times a week, we encourage each other, we celebrate each other’s successes. This support system is invaluable. When you know people believe in you and are cheering for you, you believe in yourself more too.*

Participants emphasized that observing peers successfully engage in tennis provided powerful vicarious experiences—another key source of self-efficacy according to Bandura’s theory. Seeing similar others succeed increased participants’ beliefs that they too could achieve similar outcomes. Participant P11 described:

*When I see other people my age playing well, staying active, it inspires me. I think, “If they can do it, so can I.” It’s especially motivating when I see someone older than me playing strong tennis. It shows me what’s possible.*

Unlike social support, vicarious experience works through observation. Watching a peer of similar age or ability succeed leads one to believe they can do the same, making the age-matched environment of a tennis club particularly conducive to self-efficacy [9].

The social comparison processes within tennis communities appeared to be largely positive and motivating rather than discouraging. Participants attributed this to the supportive culture within their tennis groups. Participant P10 noted:

*In our tennis club, we don’t just compete—we support each other. When someone improves or achieves something, we all celebrate. There’s no harsh judgment if you make mistakes. This positive environment makes you feel confident to try your best.*

Several participants described how the social obligations and commitments associated with tennis participation helped maintain their engagement, which in turn sustained the benefits for self-efficacy. Participant P4 explained:

*Knowing that my tennis partners are expecting me motivates me to show up, even on days when I’m not feeling energetic. This regular participation keeps me active and engaged. And the more consistently I participate, the more capable I feel.*

The intergenerational aspects of tennis also emerged as important. Several participants described playing with or observing younger players, which challenged age-based stereotypes and enhanced their sense of capability. Participant P5 reflected:

*Sometimes I play with younger players, and I can hold my own against players in their 50s or even 40s. This breaks down the idea that being in your 70s means you’re limited. It shows that age is just a number if you stay active and engaged.*

Participants also described how the social networks formed through tennis extended beyond the court, providing broader life support and enhancing overall well-being. Participant P8 explained:

*My tennis friends and I support each other in all aspects of life, not just tennis. We share advice about health, we help each other with various problems, we socialize outside of tennis. This comprehensive support network makes me feel more confident in managing my life.*

The social bonds formed through tennis also extended to family relationships, further reinforcing a sense of shared identity and belonging. Participant P11 described how tennis had become central not only to his personal identity but to that of his entire household:

*We call ourselves a tennis family. If you cannot play tennis, you are not recognized as a family member. My son and daughter, who now live in Australia, play tennis there and even won a doubles tournament recently. When I visit, we play together. Tennis gives us a shared language no matter where we are.*

Beyond family, the social dimension of tennis was described as a remedy for the companionship deficit that often accompanies aging. Participant P8 further emphasized the role of tennis as a source of meaningful peer relationships in later life:

*As you get older, you have no friends. Tennis gives you a companion to talk to, someone to hit with, and a partner who can comfort you when you go through difficulties. That kind of partnership is something you cannot easily find elsewhere at this age.*

### 3.3. Theme 3: Physical Vitality as Confidence Foundation

This theme captures how the physical health benefits derived from tennis participation served as a foundation for broader self-efficacy beliefs. Participants consistently described how improved physical fitness, functional capacity, and health outcomes enhanced their confidence in their ability to maintain independence and manage daily activities.

Participants emphasized that tennis provided comprehensive physical conditioning, including cardiovascular fitness, strength, flexibility, and coordination. These physical improvements translated directly into enhanced functional capacity and confidence. Participant P1 described:

*Since playing tennis regularly, I’m much stronger and more energetic. I can do things that many people my age struggle with—carrying heavy groceries, climbing stairs without getting winded, keeping up with my grandchildren. This physical capability gives me tremendous confidence.*

Several participants compared their physical condition with that of peers who did not engage in regular physical activity, noting that this comparison reinforced their sense of capability and the value of their tennis participation. Participant P4 explained:

*Many of my friends who don’t exercise complain about aches, pains, and fatigue. They struggle with activities that I find easy. Seeing this difference makes me appreciate what tennis has done for me and makes me confident that I’m aging well.*

The maintenance of physical independence emerged as a critical factor linking physical vitality to self-efficacy. Participants expressed a strong determination to remain independent and viewed their physical fitness from tennis as essential for achieving this goal. Participant P6 stated:

*Independence is everything to me. I don’t want to be a burden on my children or need assistance for basic activities. Tennis keeps me physically capable of taking care of myself. This independence gives me confidence.*

Participants also described how physical improvements from tennis enhanced their confidence in managing health challenges and preventing disease. Participant P1 explained:

*I have some chronic conditions—high blood pressure, early arthritis. But tennis helps me manage these. My doctor says my cardiovascular health is excellent for my age. Knowing that I’m actively protecting my health through tennis makes me feel in control.*

The pain management and functional benefits of tennis were particularly salient for participants experiencing age-related physical challenges. Participant P6 described:

*I used to have significant back pain and stiffness. Since playing tennis regularly, these problems have greatly improved. The movement, the stretching, the strengthening—it all helps. Being relatively pain-free gives me confidence to engage in activities I might otherwise avoid.*

Several participants described how their physical capability from tennis challenged their own and others’ age-based expectations, enhancing their sense of self-efficacy. Participant P7 reflected:

*At 75, people expect you to be frail and limited. But I’m not. I’m active, strong, capable. Tennis has kept me this way. When people are surprised by what I can do at my age, it reinforces my belief that I’m not defined by my age—I’m defined by what I do.*

Participants’ self-directed approach to physical maintenance reflects a strong sense of personal agency over one’s health trajectory—a key marker of physical self-efficacy. Yet participants also acknowledged the gradual physical limitations that accompany advanced age. Participant P1 noted that while the commitment to tennis remained unwavering, the body’s response had begun to signal natural constraints:

*No matter how hard you want to try, the muscles and joints weaken, and even though you want to try hard, it does not work out the way it used to. Recently that kind of phenomenon has been appearing very distinctly. As anyone gets older, playing tennis naturally becomes somewhat restricted.*

Such candid acknowledgment of physical decline did not, however, undermine participants’ overall sense of efficacy. Rather, it reflected a realistic appraisal of aging that coexisted with continued confidence in one’s capacity to remain active and engaged.

### 3.4. Theme 4: Mental Resilience and Cognitive Engagement

This theme encompasses the psychological and cognitive benefits of tennis participation that contribute to self-efficacy. Participants described how tennis provided stress relief, cognitive stimulation, and opportunities for mental challenge that enhanced their psychological well-being and confidence in their mental capabilities.

Participants consistently described tennis as a form of stress relief and emotional regulation. The immersive nature of tennis play appeared to provide respite from worries and negative emotions. Participant P2 explained:

*When I’m on the tennis court, I’m completely focused on the game. All my worries and stresses disappear. This mental break is incredibly refreshing. After playing, I feel renewed and better able to handle whatever challenges I’m facing.*

The cognitive demands of tennis—including strategic thinking, quick decision-making, and attention—were valued by participants as mental exercise that maintained cognitive function. Participant P9 described:

*Tennis is not just physical—it’s highly mental. You have to anticipate your opponent’s moves, plan your strategy, make split-second decisions. This constant mental engagement keeps your mind sharp. I believe tennis has helped me maintain my cognitive abilities as I age.*

Several participants described how the mental challenges of tennis built psychological resilience and confidence in their ability to handle complex situations. Participant P3 explained:

*Tennis teaches you mental toughness. You have to stay focused under pressure, recover from mistakes, maintain confidence even when losing. These mental skills transfer to other areas of life. I feel more mentally resilient and capable because of tennis.*

The sense of purpose and engagement provided by tennis emerged as an important contributor to psychological well-being and self-efficacy. Participant P2 reflected:

*Tennis gives me something to look forward to, something to work toward. This sense of purpose is vital. Many older adults feel aimless or unproductive. But I have goals in tennis—improving my game, competing in tournaments. This purposeful engagement makes me feel vital and confident.*

Participants also described how success in managing the mental challenges of tennis enhanced their confidence in their cognitive abilities more broadly. Participant P8 stated:

*When I successfully execute a complex strategy in tennis or make good decisions under pressure, it proves to me that my mind is still sharp. This confidence in my mental abilities extends beyond tennis—I feel confident learning new things, solving problems, staying mentally active.*

Several participants described how tennis provided a healthy outlet for competitive drives and achievement motivation that might otherwise diminish in retirement. Participant P5 explained:

*After retiring, I missed the challenge and competition of work. Tennis fills that void. I can still compete, still strive for excellence, still achieve. This continued engagement with challenge and achievement is psychologically important. It maintains my drive and confidence.*

The mindfulness and present-moment awareness cultivated through tennis play also emerged as beneficial for mental well-being. Participant P10 described:

*Tennis requires complete presence. You can’t be thinking about the past or future—you have to be fully in the moment. This mindful engagement is calming and centering. It’s helped me develop greater mental clarity and emotional balance.*

The stress-restorative function of tennis was described with particular vividness by participants who had engaged in the sport across decades of life’s challenges. Participant P4 articulated how tennis served as a psychological reset mechanism:

*When there is something difficult, or when there is some worry, through tennis I am able to forget everything. After it ends, I come to have a new mindset and am able to start something new again. It is considerably helpful for stress relief, and I believe it has even helped me set new goals in life.*

This capacity of tennis to facilitate psychological renewal was reinforced by participants’ accounts of how the sport helped them navigate interpersonal tensions and self-awareness. Participant P4 also reflected on how tennis served as an unexpected mirror for self-discovery:

*Through club life, I am able to discover myself. I notice what shortcomings I have, and how certain behaviors make others less comfortable. That becomes an opportunity for me to correct them. The tennis community reveals your weaknesses and gives you a chance to grow.*

Together, these accounts underscore how the psychological benefits of long-term tennis participation extend well beyond simple stress reduction, encompassing self-reflection, interpersonal learning, and the continual renewal of motivation—all of which contribute to a deepened and sustained sense of efficacy in later life.

## 4. Discussion

This qualitative study explored the relationship between long-term participation in tennis and self-efficacy in older South Korean adults. Four themes emerged from interviews with participants regarding how tennis contributed to enhanced self-efficacy in late life. Participants’ tennis experiences were related to their mastery, social support, physical health, and psychological well-being. Implications for self-efficacy in late life and practical strategies for promoting physical activity to foster healthy aging are discussed.

### 4.1. Mastery Experiences and Progressive Achievement

The first theme was entitled ‘Mastery Through Progressive Achievement’. Bandura [9,34] described mastery experiences as the most effective source of enhancing self-efficacy. Since tennis is a highly skill-based sport, many of the participants’ comments pertained to their own progression through the various skill levels and their improved performance relative to others, enabling them to compete successfully.

Research has consistently found that physical activity can enhance self-efficacy and that activities that provide high feedback and challenge are most effective in achieving this outcome [16]. Although many forms of physical activity become routine for adults and cease to offer opportunities for challenge, tennis is an activity that offers continued skill development and accomplishment for adults at many stages of the life span. Additionally, given the norm of loss and decline for many aspects of adult life, the opportunity for continued growth and mastery across varying levels of play may play a particular role in enhancing self-efficacy in the older adult years.

The concept of generalized self-efficacy refers to the extent to which individuals believe in their overall competence across a variety of situations [35]. Research has found that support exists for this concept, as players reported how the confidence they gained from tennis generalized to other areas of their lives. While Bandura described self-efficacy beliefs as having a domain-specific focus, experience in one domain can generalize to other related domains where individuals perceive similar skills to be required to achieve success in those domains [36]. The reports of players regarding the use of their core tennis skills of resilience, persistence and problem solving in a variety of life situations present an additional domain in which success in tennis can be experienced.

Participants reported that focusing on small steps to achieve reasonable challenges in learning sports skills is a key element of quality sports skills development. Research on optimal challenge also suggests that too easy or too difficult challenges do not facilitate meaningful mastery experiences [37]. For older tennis players, self-efficacy grows when challenge matches ability, not too easy and not too hard. Being able to regulate one’s own challenge level, such as choosing suitable opponents or adjusting play style, may be key to sustaining mastery experiences over time [36]. Tennis appears to be a sport that can be played at several levels of proficiency by individuals as well as in competitive matches against others at a level of play that is reasonable for both parties.

While participation was largely positive, participants noted gradual physical limitations such as joint discomfort and slower recovery. Self-efficacy may thus be sustained through adaptive participation rather than continuous improvement.

Practically, these findings suggest that tennis programs for older adults should be developed around progressive, achievable challenges rather than fixed performance standards, enabling mastery experiences across varying skill levels and sustaining engagement as physical capacity changes.

### 4.2. Social Support and Collective Efficacy

The second theme, identified in the quotes and participant descriptions, was Social Embeddedness and Collective Efficacy. The participants’ social embeddedness in the tennis community positively impacted their self-efficacy. Time and again, participants spoke of the “positive support” they received. Observing others successfully master specific aspects of tennis and knowing others who succeeded at tennis also enhanced participants’ feelings of confidence toward tennis. Social cognitive theory suggests that vicarious experience and social persuasion can influence an individual’s self-efficacy beliefs [9].

While previous research has investigated the negative effects of social comparison in some contexts (e.g., academic attainment, body image) [38], the present study found that social comparison within tennis communities functioned as a predominantly positive mechanism through which participants perceived their self-efficacy to be reinforced. This emerged as one of the key mechanisms through which tennis participation shaped self-efficacy beliefs—consistent with the study’s aim to identify specific pathways linking long-term sport engagement to self-efficacy in later life. The cultural characteristics of tennis as experienced within the specific club settings sampled in this study, which participants described as positive and supportive, are discussed in relation to how participation in physical activity is associated with enhanced self-efficacy through social comparison [9,38].

The concept of collective efficacy—beliefs about the capabilities of a group to achieve goals—may also be relevant to understanding these findings [39]. Participants described a sense of shared capability within their tennis communities, where the group’s collective achievements and mutual support enhanced individual members’ confidence. This collective dimension of efficacy has received less attention in aging research but may be particularly important for older adults, whose individual capabilities may be enhanced through supportive social networks.

The finding that tennis-based social networks provided support extending beyond the sport itself highlights the potential for physical activity participation to address the social isolation that threatens many older adults’ well-being [7]. The regular, structured nature of tennis participation appeared to facilitate the development of meaningful relationships that provided comprehensive life support, not just sport-specific encouragement. This broader social integration may amplify the benefits of physical activity for psychological well-being and self-efficacy in later life.

The intergenerational aspects of tennis participation that emerged in this study are noteworthy. Participants described how playing with or observing younger players challenged age-based stereotypes and enhanced their sense of capability. This finding aligns with research on age stereotypes and aging, which has documented that negative age stereotypes can become self-fulfilling prophecies, diminishing older adults’ performance and self-efficacy [40]. Conversely, experiences that challenge these stereotypes—such as successfully competing with younger players—may enhance self-efficacy by contradicting limiting beliefs about aging.

However, club-based environments may not benefit everyone equally, as less competitive or physically capable individuals may feel excluded. Fostering inclusive club cultures that welcome varying ability levels is therefore essential to broadening access to these social benefits.

### 4.3. Physical Health as a Foundation for Confidence

Participants reported that a sense of physical capability, energy, and functional independence gained through tennis increased their confidence in managing everyday life. As fitness was not objectively measured, these findings reflect subjective perceptions rather than demonstrated physical changes. These findings are supported by research into physical function and self-efficacy in older age [41,42]. Consistent with these findings, participants in the present study described perceived physical vitality as closely tied to their sense of independence, which emerged as a prominent theme throughout the data.

Physical capability was perceived to be important in enabling independent living, and tennis was perceived to be important in helping to maintain physical capability, thereby supporting independence [43].

Participants’ descriptions of how their perceived physical vitality from tennis supported their confidence in managing health challenges align with the concept of health self-efficacy—beliefs about one’s ability to manage health and prevent disease [9]. Research has documented that health self-efficacy predicts health behaviors, health outcomes, and quality of life in older adults [44]. The findings of this study suggest that regular physical activity like tennis may support health self-efficacy by fostering a perceived sense of agency over one’s health and physical functioning.

Participants’ reports of pain relief and functional improvement align with research on physical activity and chronic pain [44,45], though pain and physical capacity were not objectively assessed. Rather, the subjective sense of managing physical challenges through tennis appeared to generate mastery experiences that supported self-efficacy. This suggests that emphasizing perceived functional progress may be a meaningful, confidence-building goal in physical activity programs for older adults.

Participants felt that staying physically active through tennis challenged the idea that aging means inevitable decline. By seeing themselves as capable and active, they came to believe that getting older does not have to mean becoming weak or limited. This resistance to age-based stereotypes may be psychologically protective, enhancing self-efficacy by expanding perceptions of what is possible in later life [46].

These findings support investing in community tennis facilities and affordable programs for older adults, especially those focused on maintaining independence and managing long-term health conditions.

### 4.4. Psychological Benefits and Cognitive Engagement

The fourth theme, “Mental Resilience and Cognitive Engagement,” captured how tennis enhanced the participants’ perception of self-efficacy, highlighting how tennis reduced stress, stimulated the mind, required mental effort to practice, and gave a sense of purpose to training, thereby enhancing psychological well-being. Physical activity has been shown to have many positive effects on mental health, acting as an effective distraction from stress and worry [47]. Although it is unlikely that many people play tennis without thinking about winning or losing, becoming immersed in play can be an absorbing experience that facilitates disengagement from everyday concerns. This can have an indirect effect on self-efficacy by maintaining a positive affect that is associated with increased confidence and motivation [48].

Participants consistently highlighted the cognitive benefits associated with tennis participation. A number of research studies have investigated the relationship between physical activity in older age and cognitive function, generally finding that physical activity can either maintain or enhance cognitive function [49,50]. By being aware of the potential cognitive benefits of playing tennis, older adults are likely to enhance their cognitive self-efficacy, perceiving themselves as mentally capable and competent.

Mental toughness and psychological resilience, as they relate to tennis, were seen by participants to be important concepts related to the play of the sport, as noted in the literature on sport, physical activity, and psychological resilience [51]. It should be acknowledged that much of this literature is derived from competitive or elite athlete populations and may not generalize directly to older recreational players. However, the participants in this study themselves described cognitive demands—such as managing pressure, recovering from errors, and sustaining focus—as meaningful aspects of their tennis experience, lending ecological validity to the application of resilience concepts in this context. The mental aspects of tennis, such as concentration, bouncing back from adversity, and perseverance, can teach players psychological skills that transfer to other life situations. This is particularly relevant to older adults who are faced with many changes, losses, and other age-related challenges as they age.

The sense of purpose and meaningful engagement provided by tennis participation emerged as an important contributor to psychological well-being and self-efficacy. Research has consistently documented the importance of purpose and meaning for well-being in later life [52]. Through tennis, the participants found purpose in physical activity as they set goals, mastered new skills, and engaged in meaningful interactions with others [51,52].

Unlike walking or stretching, tennis combines skill development, strategic thinking, and social interaction in a single activity, offering simultaneous opportunities for mastery and social reinforcement. This may explain why tennis is particularly effective in sustaining self-efficacy across the lifespan.

### 4.5. Limitations and Future Research

The study has some limitations. First, the study is specific to tennis, and therefore, the findings must be interpreted with caution in terms of their generalizability to other physical activities. However, given that tennis involves a combination of physical, skill-based, and social elements, it is reasonable to conclude that the underlying mechanisms may be applicable to other activities. Second, the study is culturally specific and therefore the findings may contain elements reflective of Korean culture. However, it is anticipated that the underlying mechanisms should have cross-cultural relevance and applicability to other populations. Third, the all-male sample of 11 limits transferability, as gender moderates physical activity motivation and self-efficacy development. Fourth, recruitment through tennis clubs in a limited region may have introduced selection bias toward active, socially engaged individuals. Future research should include female participants, recruit from diverse regions, and incorporate non-club members to strengthen generalizability. These findings should therefore be interpreted as context-specific insights from a particular group rather than conclusions applicable to all older adults. Tennis may thus be understood not merely as one physical activity among many, but as a multidimensional platform that distinctively supports sustained self-efficacy in later life.

## 5. Conclusions

This qualitative study examined the relationship between long-term participation in tennis and self-efficacy among older adult Korean tennis players. The findings demonstrated that participation in tennis supports self-efficacy through mastery experiences (skill-based tennis performance), social support (a supportive community of tennis players), physical health (positive physical changes associated with healthy aging), and psychological benefits (enhanced cognitive function and mental health). Thus, tennis may serve as an effective intervention strategy for meeting some of the needs for healthy aging.

The results contribute to the theoretical understanding of how self-efficacy develops and is maintained in later life, highlighting the importance of activities that provide opportunities for continued growth, achievement, social connection, and meaningful engagement. Practically, the findings suggest that tennis programs designed for older adults, emphasizing skill development, social support, and long-term engagement, can be valuable tools for promoting self-efficacy and overall well-being in aging populations.

Future research should examine these relationships longitudinally, investigate the experiences of diverse populations of older adults—including female participants to address the gender imbalance of the present study—compare tennis to other forms of physical activity, and develop and test interventions based on the mechanisms identified in this study. Additionally, research should explore potential moderators and mediators of the relationship between tennis participation and self-efficacy, including personality factors, social support, and cultural contexts.

As populations worldwide age rapidly, identifying effective strategies for promoting healthy aging becomes increasingly critical. This study suggests that long-term engagement in appropriately designed physical activities like tennis can play an important role in maintaining self-efficacy, a key psychological resource for aging well. Supporting older adults’ participation in such activities should be a priority for public health initiatives aimed at promoting healthy aging.

## Figures and Tables

**Table 1:** Participant demographics and tennis participation characteristics.

ID	Gender	Age	Years of Tennis	Weekly Participation (Sessions/Week)	Region
P1	Male	68	15	3	Chungbuk
P2	Male	72	18	3	Gyeonggi
P3	Male	65	15	2	Chungbuk
P4	Male	70	20	3	Gyeonggi
P5	Male	74	22	2	Chungbuk
P6	Male	67	17	3	Chungbuk
P7	Male	75	25	2	Gyeonggi
P8	Male	69	25	3	Chungbuk
P9	Male	71	18	2	Gyeonggi
P10	Male	66	15	3	Chungbuk
P11	Male	73	21	2	Gyeonggi

## Data Availability

The data presented in the study are available on request from the corresponding author due to confidentiality and privacy restrictions.
